# BST Stimulation Induces Atrophy and Changes in Aerobic Energy Metabolism in Rat Skeletal Muscles—The Biphasic Action of Endogenous Glucocorticoids

**DOI:** 10.3390/ijms21082787

**Published:** 2020-04-17

**Authors:** Mateusz Jakub Karnia, Dorota Myślińska, Katarzyna Patrycja Dzik, Damian Józef Flis, Magdalena Podlacha, Jan Jacek Kaczor

**Affiliations:** 1 Department of Bioenergetics and Nutrition, Gdansk University of Physical Education and Sport, Kazimierza Górskiego 1, 80-336 Gdansk, Poland; mateusz.karnia@awf.gda.pl (M.J.K.); katarzyna.dzik@awf.gda.pl (K.P.D.); damian.flis@awf.gda.pl (D.J.F.); 2 Department of Animal and Human Physiology, Faculty of Biology, University of Gdansk, 80-308 Gdansk, Poland; dorota.myslinska@biol.ug.edu.pl; 3 Department of Molecular Biology, Faculty of Biology, University of Gdansk, 80-308 Gdansk, Poland; magdalena.podlacha@biol.ug.edu.pl

**Keywords:** brain stimulation, glucocorticoids, mineralocorticoid receptor, glucocorticoid receptor, chronic stress, mitochondria

## Abstract

(1) The primary involvement in stress-induced disturbances in skeletal muscles is assigned to the release of glucocorticoids (GCs). The current study aims to investigate the impact of the biphasic action of the chronic stress response (CSR) induced by the electrical stimulation of the bed nucleus of the stria terminalis (BST) effects on muscle atrophy and aerobic energy metabolism in soleus (SOL) and extensor digitorum longus (EDL) muscles. (2) Male Wistar rats (*n* = 17) were used. The rats were divided randomly into three groups: the BST two weeks (ST2), four weeks (ST4), and the sham (SHM) electrically stimulated group. The plasma corticosterone (CORT) and irisin concentration were measured. Glucocorticoid and mineralocorticoid receptors (GR and MR), 11β-hydroxysteroid dehydrogenase type 1 and 2 (HSD11B1 and HSD11B2), atrogin-1, and insulin-like growth factor-1 (IGF-1) level were determined in SOL and EDL muscles. Citrate synthase (CS) activity was measured in both muscles. (3) We found elevated plasma concentration of CORT and irisin, raised the level of GR in SOL muscle, and the higher level of MR in both muscles in the ST4 group. The level of HSD11B1 was also higher in the ST4 group compared to the SHM group. Moreover, we observed increased activity of CS in SOL. (4) We suggest that biphasic action of the glucocorticoid induced by the CSR occurs and causes dysregulation of proteins involved in muscle atrophy and aerobic energy metabolism. Our findings potentially contribute to a better understanding of the mechanisms by which GCs and the CSR may regulate muscle atrophy and energy preservation of the red muscle.

## 1. Introduction

Stress is one of the most alarming health problems in the modern world. This explains a pressing need for explorations into the biological mechanisms and pathways linking stress and health. It is well documented that the chronic stress response (CSR) leads to extremely negative consequences and is linked to many disease states, affecting the health of many populations. Stress-induced disturbances occur through multiple biochemical and signaling pathways. However, the major involvement in this process is assigned to the pathways that determine system response to stress through the release of glucocorticoids (GCs) [1,2]. We propose the electric stimulation of the bed nucleus of the stria terminalis (BST) as the model that mimics unconscious stress in rats. Many studies have proved that BST plays a crucial role in the activation of the hypothalamus–pituitary–adrenocortical (HPA) axis during stress [3,4]. Besides, the excess high level of GCs occurs in several pathologic conditions such as diabetes, starvation, cancer, burn injuries, and depression, as well as after long-term medical treatment of synthesized GCs [5].

Under stressful or pathophysiological conditions, circulating GCs levels are greatly increased, which in turn decreases the rate of protein synthesis and rises proteolysis to generate amino acids that serve as precursors for hepatic gluconeogenesis. In skeletal muscles, it leads to the two main adverse effects: firstly, the development of oxidative stress, and secondly, skeletal muscle atrophy and muscle weakness [6]. On the other hand, GCs play a biphasic role on muscle and neuronal mitochondrial dynamic, demonstrating that at a low level, they potentiate; however, at chronic high level, GCs attenuate mitochondrial energy metabolism, respectively [7].

Mounting data indicate that the effects induced by GCs may be caused and regulated independently, at multiple levels of control, by the glucocorticoid receptor (GR) and activity of 11β-hydroxysteroid dehydrogenase type 1 (HSD11B1) both in vitro and in vivo. The GCs lead their signal mainly through the intracellular GR; however, they may also act through different mechanisms. The major action is to regulate the transcription of its primary target genes through genomic glucocorticoid response elements (GREs) by directly binding to DNA or tethering onto other DNA-binding transcription factors. These GR primary targets trigger physiological and pathological responses of GCs [6]. However, the GC bioavailability and action depend not only upon circulating levels or GR content but also on tissue-specific intracellular metabolism by HSD11B1. Essential metabolic tissues, including liver, adipose tissue, and skeletal muscle express HSD11B1, whose function is to convert inactive cortisone to cortisol or corticosterone (CORT) [8].

The recent study shows the presence and the activity of the mineralocorticoid receptor (MR) in skeletal muscle [9]. MR and GR exhibit cross-reactivity with endogenous GCs, which have the same or even higher affinity to MR than GR (depending on the tissue). As a consequence of the high homology with GR, MR is activated by both mineralocorticoids (aldosterone, deoxycorticosterone) and GCs [9]. Data from the past several years have assigned a specific role for the MR in mediating oxidative stress development. Furthermore, data indicate that ALD/MR-dependent NADPH induces superoxide production with simultaneous increasing serum 8-isoprostane (8-iso) and thiobarbituric acid reactive substances (TBARs) levels [10,11].

The novel report presents that the skeletal muscles may influence adaptation to psychological stress [12], and the central role is assigned to the peroxisome proliferator-activated receptor gamma coactivator 1-alpha–fibronectin type III domain-containing protein 5–brain-derived neurotrophic factor (PGC-1α-FNDC5-BDNF) pathway. Irisin, the PGC-1α-dependent myokine, classically is secreted by skeletal muscle during exercise [13]. However, both GCs and GR might be considered as a positive regulator of FNDC5 expression [14]. Therefore, the question remains whether GCs may cause irisin augmentation as a consequence of the CSR. In particular, irisin flux is higher during high-intensity exercise than during low-intensity exercise, which is accompanied by increased cortisol level in high-intensity efforts [15].

In the present study, we aim to determine whether the CSR induced by the electrical stimulation of the BST effects on muscle atrophy and aerobic energy metabolism in soleus (SOL) (which contains predominantly slow-twitch fibers [16]) and extensor digitorum longus (EDL; containing maximum 10% of type I fibers [16]) muscles of the rat. Therefore, the study investigates the impact of the biphasic action of the CSR on the GR, MR protein content, and the HSD11′s B1 and B2 activity as well as mitochondrial function in both muscles. We presume that both augmented receptors and HSD11′s B1 and B2 activity are associated with muscle atrophy. We hypothesize that as a result of the biphasic action of GCs during the CSR, muscle atrophy in both muscles will be observed. However, on the other hand, GCs/insulin-like growth factor 1 (IGF-1) pathway will potentially play protective action in the red muscle manifested by the higher mitochondrial CS activity.

## 2. Results

### 2.1. Plasma CORT

Lately, we demonstrated on the same experimental model that rats’ plasma CORT level after the BST stimulation significantly increased in the four weeks stimulated group (ST4) group as compared with the two weeks stimulated (ST2) and sham-stimulated (SHM) groups [17]. The results are summarized in Table 1.

### 2.2. GR and MR Content in Skeletal Muscles

**Figure 1:**
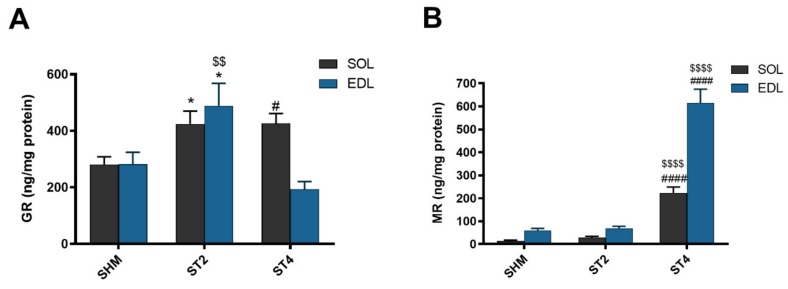
The level of glucocorticoid receptor (GR) (**A**) and mineralocorticoid receptor (MR) (**B**) in soleus (SOL) and extensor digitorum longus (EDL) muscles. Results were expressed as mean ± SEM, sham-stimulated (SHM; *n* = 6), two weeks stimulated (ST2; *n* = 6), four weeks stimulated (ST4; *n* = 5). * *p* < 0.05 SHM vs. ST2, ^#^ *p* < 0.05 SHM vs. ST4, ^$$^ *p* < 0.01 ST2 vs. ST4, ^$$$$^ p < 0.0001 ST2 vs. ST4, ^####^ p < 0.0001 SHM vs. ST4.

Muscle GR content was the highest in the ST4 group in SOL muscle (426.63 ± 77.79 ng/mg protein), and it was significantly lower in the SHM group (280.58 ± 67.39 ng/mg protein; *p* < 0.05). Moreover, the GR concentration was also significantly higher in the ST2 group when compared to the SHM group (424.86 ± 109.46 ng/mg protein; *p* < 0.05). In contrast to the SOL muscle in the EDL muscle, the GR level was the lowest in the ST4 group, and it was 193.88 ± 60.31 ng/mg protein. Also, the highest concentration of GR was observed in the ST2 group (489.49 ± 192.72 ng/mg protein), and the differences were statistically significant vs. both the SHM (282.81 ± 101.32 ng/mg protein) and ST4 groups (*p* < 0.05; Figure 1A).

The muscular concentration of MR was the highest in the ST4 group, both in SOL and EDL muscles. In SOL, it was 222.48 ± 58.24, 28.98 ± 13.02 and 14.49 ± 8.10 ng/mg protein in the ST4, ST2, and SHM groups, respectively (*p* < 0.001). Also, in the EDL muscle, the concentration of MR was elevated approximately ninefold in the ST4 group (614.34 ± 135.56) as compared to the SHM (59.38 ± 22.80) and ST2 (68.88 ± 22.65 ng/mg protein) groups (*p* < 0.001; Figure 1B).

### 2.3. HSD11B1 and HSD11B2 Content in Skeletal Muscle

**Figure 2:**
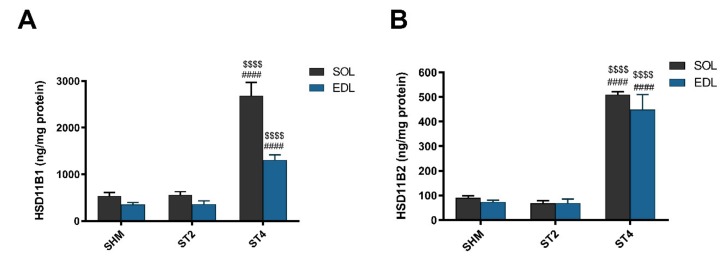
The level hydroxysteroid dehydrogenase type 1 (HSD11B1) (**A**) and hydroxysteroid dehydrogenase type 2 (HSD11B2) (**B**) in SOL and EDL muscles. Results were expressed as mean ± SEM, SHM (*n* = 6), ST2 (*n* = 6), ST4 (*n* = 5). ^$$$$^ *p* < 0.0001 ST2 vs. ST4, ^####^ *p* < 0.0001 SHM vs. ST4.

The level of HSD11B1 was the highest in the ST4 group in both muscles. In the SOL muscle, there were 2686.57 ± 629.21 in the ST4, 559.11 ± 161.63 in the ST2, and 533.53 ± 187.49 ng/mg protein in the SHM groups, respectively (*p* < 0.001). A similar observation was made in the EDL muscle, and the values were 1308.83 ± 243.08 in the ST4, 360.58 ± 176.06 in the ST2, and 352.87 ± 104.76 ng/mg protein in the SHM groups, respectively (*p* < 0.001; Figure 2A).

Also, the muscle HSD11B2 level was the highest in the ST4 group when compared to the SHM and ST2 groups in both muscles. The differences between groups were statistically significant, and in details in the SOL, it was 509.73 ± 25.91, 67.82 ± 26.50, and 90.48 ± 19.81 ng/mg protein in the ST4, ST2, and SHM groups, respectively (*p* < 0.001). In EDL the highest level of HSD11B2 in the ST4 group was observed (448.85 ± 135.96 ng/mg protein), and the differences were statistically significant when compared the ST2 and SHM groups (ST4 vs. SHM *p* < 0.001, and ST4 vs. ST2 *p* < 0.001; Figure 2B).

### 2.4. Marker of Muscle Atrophy and IGF-1 Content

**Figure 3:**
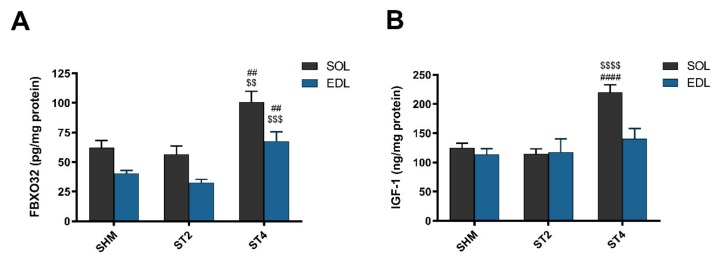
The level atrogin-1 (FBXO32) (**A**) and insulin-like growth factor-1 (IGF-1) (**B**) in SOL and EDL muscles. Results were expressed as mean ± SEM, SHM (*n* = 6), ST2 (*n* = 6), ST4 (*n* = 5). ^##^ *p* < 0.01 SHM vs. ST4, ^$$^ *p* < 0.01 ST2 vs. ST4, ^$$$^ *p* < 0.001 ST2 vs. ST4, ^$$$$^ *p* < 0.0001 ST2 vs. ST4, ^####^ *p* < 0.0001 SHM vs. ST4.

After four weeks of rat brain stimulation, the marker of muscle atrophy was elevated in the ST4 group when compared to the ST2 and SHM groups in both muscles. Atrogin-1 (FBXO32) was higher in SOL muscle, and the values were 100.86 ± 20.12 in the ST4, 56.52 ± 16.95 in the ST2, and 62.20 ± 13.49 pg/mg protein in the SHM groups, respectively (*p* < 0.01). In the EDL muscle, significant differences were observed between the ST4 and the SHM groups (*p* < 0.01; Figure 3A) and between the ST4 and the ST2 groups (*p* < 0.001). The average level of atrogin-1 was 67.77 ± 17.60, 32.70 ± 6.42, and 40.61 ± 5.40 pg/mg of protein in the ST4, ST2, and SHM groups, respectively.

In SOL, IGF-1 concentration was the highest in the ST4 group, 220.07 ± 29.15, and it was significantly lower in the ST2 (114.74 ± 19.19 ng/mg protein) and the SHM (124.83 ± 19.64 ng/mg protein) groups (*p* < 0.001; Figure 3B). We did not observe any statistically significant differences between the groups in the EDL muscle, and the values were 140.49 ± 38.99 in the ST4, 117.63 ± 55.69 in the ST2, and 113.55 ± 24.23 ng/mg protein in the SHM groups, respectively.

### 2.5. CS activity in Skeletal Muscle and Irisin Concentration in Plasma

**Figure 4:**
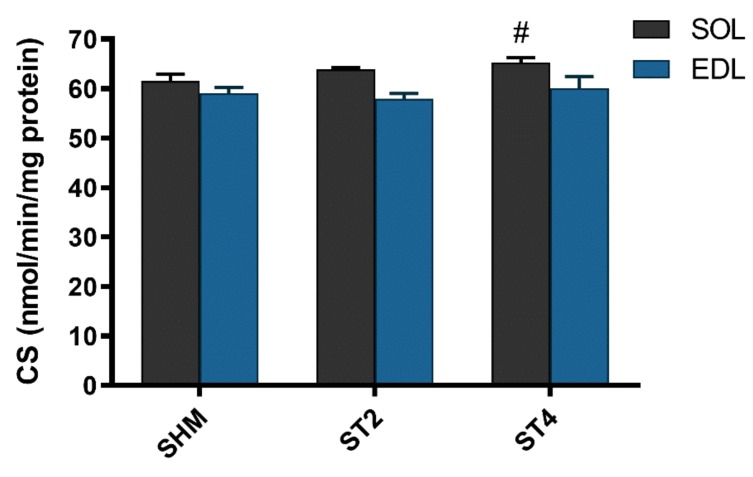
The citrate synthase (CS) activity in SOL and EDL muscles. Results were expressed as mean ± SEM, SHM (*n* = 6), ST2 (*n* = 6), ST4 (*n* = 5). ^#^ *p* < 0.05 SHM vs. ST4.

The activity of CS was not different in the EDL muscle after brain stimulation. Nevertheless, we observed statistically significant differences in the SOL muscle between the ST4 (65.30 ± 2.14) and the SHM (61.54 ± 3.30 nmol/min/mg of protein) groups (*p* < 0.05; Figure 4).

**Figure 5:**
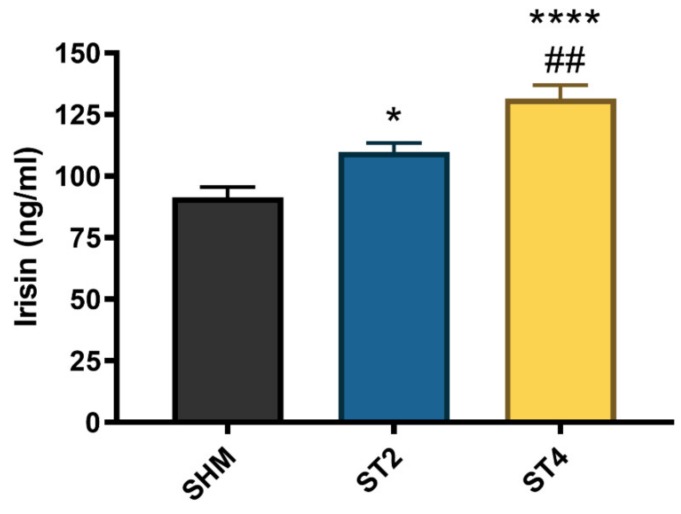
The plasma irisin level. Results were expressed as mean ± SEM, SHM (*n* = 6), ST2 (*n* = 6), ST4 (*n* = 5). * *p* < 0.05 SHM vs. ST2, ^##^ *p* < 0.01 ST2 vs. ST4, **** *p* < 0.0001 SHM vs. ST4.

The irisin level significantly differed between the groups, and the highest concentration was observed in the ST4 (131.58 ± 11.86 ng/mL) as compared to the ST2 and SHM (109.96 ± 8.57 and 91.41 ± 10.30 ng/mL, respectively). Moreover, the irisin level was also significantly higher in the ST2 group than in the SHM group (*p* < 0.05, Figure 5).

## 3. Discussion

We demonstrate that the biphasic action of CORT and the CSR caused the skeletal muscle atrophy measured by atrogin-1 level in both types of skeletal muscles with the simultaneously elevated level of HSD11B1, HSD11B2, and MR. In addition, we noticed a higher level of GR after 4 weeks in the CSR group as compared to the SHM group but solely in the red muscle. On the other hand, we found a higher IGF-1 level and an elevated CS activity only in SOL muscle. Additionally, the highest concentration of irisin in blood in the ST4 group was observed. Our findings have the potential to contribute to a complete understanding of mechanisms by which CORT and the CSR may regulate muscle atrophy, as well as preservation in the red, and devastation in the white muscles.

According to our knowledge, this is the first preclinical work that shows the interplay between HSD11B (type 1 and 2) and GR/MR in the CSR conditions. Obtained data show that the CSR linked with the elevated CORT level [17] inducing the skeletal muscle atrophy is associated with the FOXO/atrogin-1 pathway. Our observations confirm the previously published data, which clearly shows that HSD11B1 may be a major regulator of the muscular atrophy. For instance, increased HSD11B1 activity in skeletal muscle is linked with the development of insulin resistance, a decrease of muscle mass, and elevated gene expression associated with muscle atrophy [18]. However, the opposite effect was observed in the experiment conducted on HSD11B1 knockout mice. The level of skeletal muscle atrophy markers (MuRF-1 and atrogin-1) was lower in HSD11B1 KO mice as compared to the control group after CORT administration [8].

Furthermore, Zhao and co-workers proved that reduced GR expression in C2C12 myotubes compromises GC-reduced protein degradation [19]. Also, it is known that fast-twitch muscles contain higher GR content as compared to slow-twitch muscles [20]. It implies a conviction that a white muscle is more susceptible to develop adverse effects of GCs action. Our observation of a higher level of protein oxidation in EDL, as compared to SOL muscle, in the CSR rats partially proves previous results [17]. On the other hand, our results show that in EDL, not GR but MR content is dominant, and could be a significant contributing factor in muscle devastation. Some authors have postulated that the inhibition of MR improved skeletal muscle function and pathology in DMD mice [9] and prevented sarcopenia in older people [21]. Moreover, data reported that GR–MR interactions do occur and that they have functional consequences on gene transcription, including increasing gene upregulation and downregulation. In this way, the interactions between GR and MR can enhance the magnitude of the transcriptional response to GCs [22], and this can be a partial explanation of the biphasic effects of GCs on EDL and SOL muscles.

Apart from protein degradation in skeletal muscle cells, it should be noted that many GC-induced products also can repress protein synthesis through various mechanisms. It is believed that by mainly blocking the transport of amino acids into the muscle, this inhibits the stimulatory action of insulin-like growth factor 1 (IGF-1), and the influence of amino acids on mammalian target of rapamycin (mTOR) through the induction of regulated in development and DNA damage responses 1 (REDD1) and Kruppel-like factor 15 (KLF15) [23]. Surprisingly, we did not observe an expected decrease in IGF-1 level, but we have found an elevation of the concentration of that particular protein, but only in red muscle. However, some reports show that under certain conditions, like immobilization, muscle denervation [24], or in atrophy models induced by proinflammatory cytokines [25], IGF-1 does not prevent muscle cell atrophy, and its level may stay unaltered.

Lately, it was reported that ROS generation is involved in the aging process and dysfunction in several metabolic and neurodegenerative diseases, which may be partially connected with elevated GCs level [26]. In our recent work, it was presented that the CSR induced by the electrical stimulation of the BST causes the elevated level of markers lipid and protein peroxidation in EDL muscle. However, no changes in protein oxidation but only the higher level of lipid peroxidation marker in the SOL muscle was found [17]. Furthermore, the oxidation of sulfhydryl groups likely contributes to the deactivation and degradation of mitochondrial enzymes and transport proteins [27]. Possibly, that elevated protein damage observed in white, not in red muscle, might partially explain the biphasic action of the excessive flux of endogenous GCs. However, most of the available data related to skeletal muscles have been based on models with synthetic GCs administration, rather than the induction of endogenous GCs secretion. It seems to be worth noting because there is evidence indicating that administering exogenous GCs to mimic a condition of physiological stress may not reflect a realistic condition whether circulating GCs may attain well above peak levels observed during a stress response [28].

In the present study, we found the increased activity of CS in red muscle, but not in white muscle in the CSR conditions. These findings are consistent with previous observations from animals and humans experiments. Thus, in detail, Koerts-de Lang and co-workers show that CS activity in the tibialis anterior was significantly higher in the rats treated with triamcinolone compared to the control and prednisolone-treated rats [29]. Similar effects (statistically insignificant) were observed in tibialis anterior from patients with severe COPD treated with prednisolone [30]. What is more, Weber and co-workers showed a 2.5-fold induction of cytochrome c oxidase activity in myotubes treated with dexamethasone (DEX). They presented 2-fold elevated levels of mitochondrial mRNAs and a 2-fold increase of cytochrome c oxidase activity in quadriceps muscle of rats after treatment with DEX [31]. However, several studies show a lack of abnormalities or even a decreased activity of CS [32,33] but in the deltoid and quadriceps muscles. These discrepancies may be associated with different composition of muscle fibers or with the concentration of endogenous or synthetic GCs in the mentioned studies or unknown factors. However, the current study demonstrates the partially protective effect of CORT on SOL muscle accompanying elevated CS activity in the ST4 group.

Additionally, the activity of CS in EDL did not differ between the groups, which are also consistent with the data mentioned above. It was reported that high levels of circulating GCs stimulate mitochondrial biogenesis, and that among the cell types chosen for analysis, this occurrence is rather specific for skeletal muscle [31]. Hence, the question arises of why this phenomenon only affects red muscle? We observed the elevated GR level in the stressed rats only in SOL muscle. A possible mechanism could be linked with the fact that only GR (as opposed to MR) is found in mitochondria, suggesting that GRs drive the direct actions of glucocorticoids on mitochondria [34].

Furthermore, higher CS activity in SOL muscle is necessary because as energy-demanding by mitochondria to cover the expanded ATP-turnover in this stressful condition. This higher CS activity in red muscle is associated with higher irisin flux in serum and likely with augmented PGC-1α content in skeletal muscle. Recently, in work from our laboratory, we demonstrated that a relationship between the PGC-1α and CS activity exists as an indirect marker of mitochondrial biogenesis [35]. Additionally, we observed the significantly higher level of IGF-1 but only in SOL muscle, and some researchers postulate that IGF/PI3K/AKT signaling also plays a crucial role in mitochondrial biogenesis [36].

Besides, we can also suggest that the increased activity of CS in the SOL muscle might be linked with the elevated irisin level in plasma in the ST4 and ST2 groups. Research shows that both cortisol and GR might be considered as a positive regulator of FNDC5 expression [14], and that stays in line with our results (the highest level of CORT and GR in the ST4 group). Moreover, numerous studies indicate the presence of the PGC1-α/FNDC5/Irisin pathway [37,38], and that elevation in the FNDC5 gene expression in skeletal muscles is dependent on PGC-1α [13]. The PGC-1α, in turn, is thought about a mitochondrial biogenesis modulator [39], and this can be used to explain the possible indirect mechanism of higher CS activity in red muscle in the CSR conditions.

## 4. Materials and Methods

### 4.1. Animals

The animals used in the experiment were previously described by Karnia and co-workers [17]. Briefly, male Wistar rats (*n* = 17) weighing 250–300 g were used. They were provided with a standard diet ad libitum with free access to tap water and were maintained on a 12 h light/dark cycle, temperature 22 °C, humidity 50–55%.

### 4.2. Study Design

The study design was previously described by Karnia and co-workers [17]. In brief, the habituation of the animals was conducted daily for about two weeks before the experiment to minimize stress caused by the experimental procedures. All procedures were performed between 8:00 and 12:00. The rats were divided randomly into three groups: the BST two weeks electrically stimulated group (ST2; *n* = 6), the BST four weeks electrically stimulated group (ST4; *n* = 5), and the BST sham group (SHM; operated but not stimulated, *n* = 6).

The experiments were carried out according to the European Communities Council Directive (86/609/EEC), and the protocols were approved by the Local Animal Research Ethical Committee for the Care and Use of Laboratory Animals at the Medical University in Gdansk, Poland (No 8/2010).

### 4.3. Surgery and Electrical Stimulation

Standard stereotaxic surgery and the electrical stimulation were performed following the procedure described before [40]. Briefly, two weeks after recovery from the surgery, the stimulated groups were screened for BST stimulation-induced behavior. Once determined, the current intensity was held constant throughout 14 (ST2) or 28 (ST4) consecutive days of the stimulation. The animals from the SHM group were treated in the same way as the experimental group, with the exception that no current was passed through the electrodes.

### 4.4. Animal Sacrifice, Blood and Muscle Collection

All animals were sacrificed at a required time point (one hr after the termination of the last electric stimulation). The blood samples were collected by heart puncture one hour after the last session of stimulation. The blood was centrifuged at 2000× *g* for 10 min at 4 °C. Plasma was separated and stored at −80 °C for later analysis. EDL and SOL muscles were removed from both hind limbs following the procedure described before [17].

### 4.5. Muscle Homogenization

Prior to the chemical assays, muscles were minced and homogenized in an ice-cold buffer that contained 50 mM potassium phosphate, 1 mM EDTA, 0.5 mM DTT, 1.15% KCl and 1:200 protease inhibitor (Sigma Aldrich P8340) at pH 7.4. The homogenates were then centrifuged at 750× *g* at 4 °C for 10 min. Then the supernatant was divided into serial aliquots for enzyme activity and enzyme-linked immunosorbent assay (ELISA) measurements. Samples for ELISA were additionally centrifuged at 5000× *g* for 10 min. Protein concentration was determined using the Bradford assay (Sigma, B6916) according to the manufacturer’s instructions.

### 4.6. ELISA Assays

The following ELISA kits were used in the experiment and were determined by the supplied manufacturer’s instruction:

Irisin (EK-067-16, Phoenix Pharmaceuticals, Inc., USA) was measured in plasma.

11β-Hydroxysteroid dehydrogenase type 1 (HSD11B1; E2268r), 11β-hydroxysteroid dehydrogenase type 2 (HSD11B2; E11618r), glucocorticoid receptor (GR; E1608r), mineralocorticoid receptor (MR; E0158r, EIAab, China), atrogin-1 (FBXO32; ER1518, Fine test, China) and insulin-like growth factor 1 (IGF-1; SEA050Ra, Cloud-Clone, USA) were performed in 5000× *g* supernatant of both muscles.

### 4.7. Citrate Synthase (CS) Activity

CS activity was measured in accordance to work by Dzik and co-workers [35]. In brief, 50 μL of the supernatant of SOL or EDL (4%; 750× *g*) was added to 790 μL of buffer (0.1 M Tris-HCl, 5 mM EDTA, 0.05% Triton X100, pH 8.1), plus 100 µl of freshly made DTNB (1 mM), 10 µL acetyl-CoA (10 µM), and 50 µL of freshly made oxaloacetic acid (10 mM) to initiate the reaction. The reactions were performed at 37 °C in duplicate, and absorbance was read at 412 nm in a spectrophotometer (CE9200, Cecil Instruments Limited, Cambridge, UK). CS activity was expressed as nmol/min/mg of protein.

### 4.8. Statistical Analysis

Statistical analyses were performed using a software package (Statistica v. 13.1, StatSoft Inc., Tulsa, OK, USA). One way analysis of variance (ANOVA) was performed, and a Tukey post-hoc test was used for multiple comparisons. The results are expressed as mean ± SD, *p* values less than 0.05 were considered statistically significant.

## 5. Conclusions

**Figure 6:**
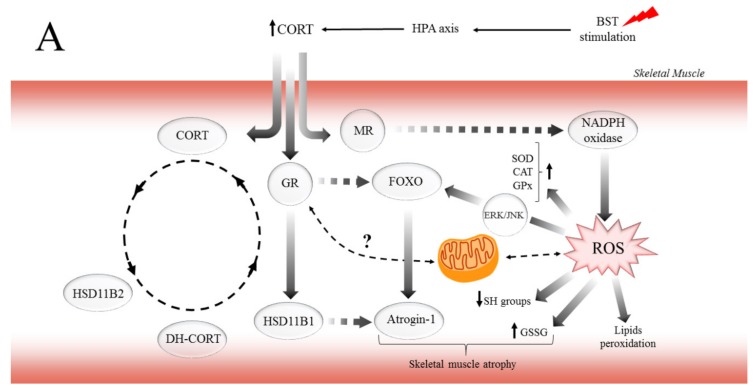

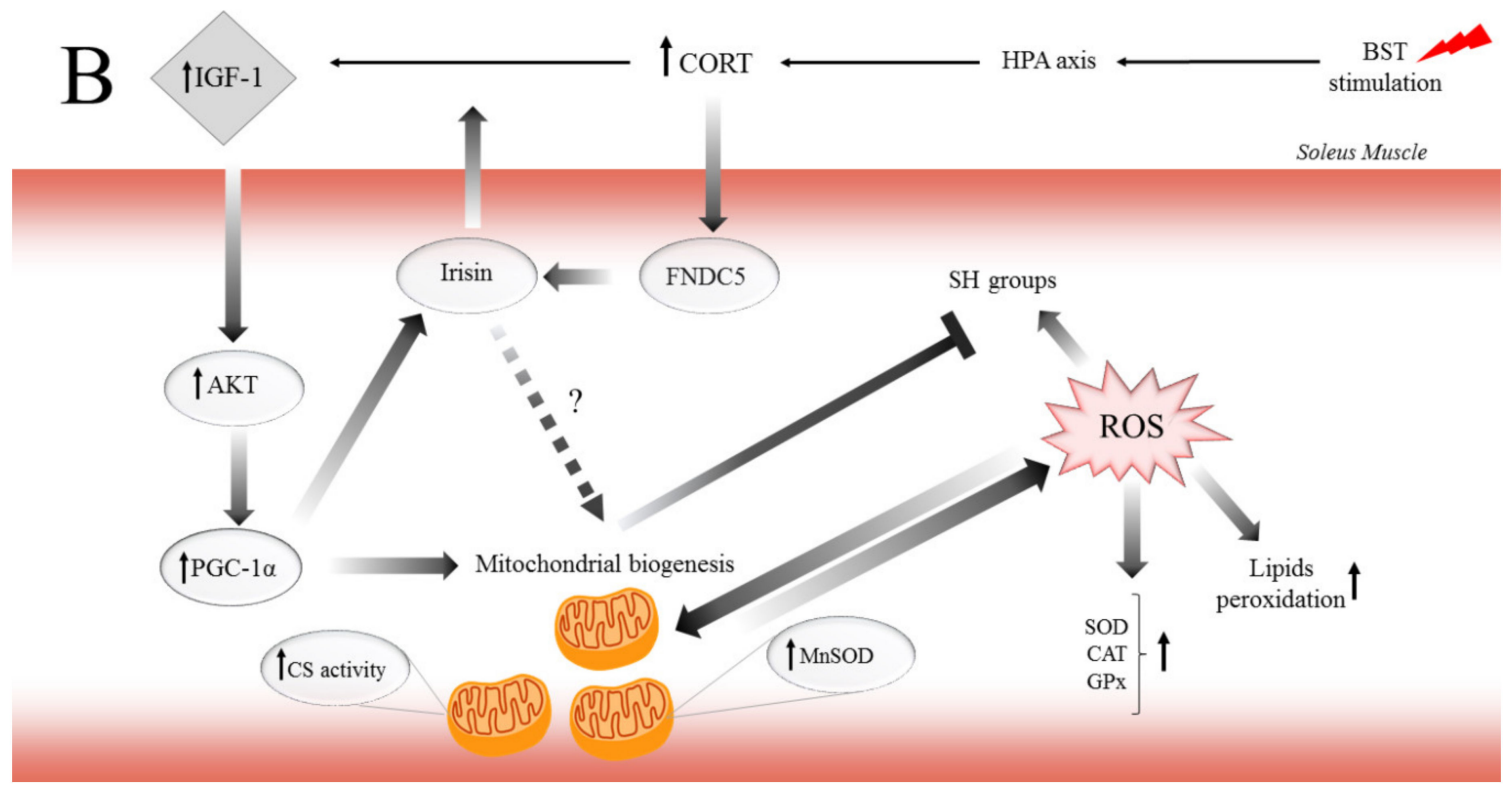
The potential mechanism of biphasic action of CORT-related changes in both (**A**) and only SOL (**B**) muscles during the CSR. (**A**) The BST stimulation mimics the CSR condition and activates the hypothalamus–pituitary–adrenocortical (HPA) axis, which results in CORT secretion. Increased circulating CORT level triggers signaling cascades, including the action of GR (thereby arising in HSD11B1 and fibronectin type III domain-containing protein 5 (FNDC5)/irisin expression), and MR (which might activate NADPH oxidase, causing the ROS generation). Furthermore, GR induces skeletal muscle atrophy in a direct (through FOXOs), and indirect (through HSD11B1-dependent atrogin-1/MURF) mechanism. Additionally, the oxidative damage of macromolecules (decreased level of SH groups, increased lipids peroxidation) occurs with simultaneous activation of enzymatic defense mechanisms (increased activity of SOD, CAT, and GPx). (**B**) In contrast to the adverse changes triggered by the CSR, the level of IGF-1 increased in red muscle and possibly induced the mitochondrial biogenesis through the IGF/PI3K/AKT pathway. Additionally, we observed increased irisin flux, and that protein traditionally is supposed to be a PGC-1α-dependent myokine; however, GR is also a positive regulator of FNDC5 gene expression. We suggest that irisin acts not only in an endocrine but also in an autocrine manner. Hence, irisin also promotes mitochondrial biogenesis in (red) skeletal muscle (manifested, i.e., by increased CS activity) and could be a part of the protective mechanism in red muscle.

In conclusion, this study demonstrates for the first time that CORT and the CSR exert major, biphasic effects on muscle atrophy and preservation of muscle devastation in red muscle. Also, to regulate aerobic energy metabolism, there may well be other mechanisms operative in the biphasic effects that GCs exerted on the muscle function. For instance, the dependent concentration–time impact of CORT on MR and GR may explain the biphasic effects of GCs on the pathophysiological role in the different types of skeletal muscle. Moreover, consistent with the findings presented here, previous work showed that elevated CORT induced lipid and protein peroxidation by ROS generation, especially in EDL muscle. On the other hand, it is possible that through GCs/IGF-1 pathway, GR mediates protective effects via different mechanisms in SOL muscle. While the different effects of CORT on MR and GR are undoubtedly crucial for various functions, the regulation of mitochondria function by GCs provides new insights to explain the biphasic effects of GCs in muscles function. Likewise, we propose the potential mechanisms of how GCs and the CSR regulate critical metabolic pathways associated with muscle atrophy (Figure 6A) and preservation of SOL muscle function (Figure 6B) as energy-demanding by mitochondria to cover the expanded ATP-turnover. The possibility that the GCs/IGF-1 pathway, which enhances the function of mitochondria, may be useful in countering the deleterious effects of excessive GCs flux observed in the CSR is an exciting prospect for future investigation. Furthermore, this also raises the intriguing problem-related in the regulation and inhibition of GR and MR action or HSD11B1 activity, as adjunctive therapy to limit the adverse effects associated with GCs excess.

### Study Limitation

In the current study, the FOXOs and PGC-1α content would give a broader and more precise picture of the described mechanisms. However, we were limited by the amount of biological material, and we had to make the most reasonable choice of protein to be determined. According to work from our laboratory [35], the atrogin-1 level was strongly related to FOXO content in humans’ skeletal muscles. Also, the relationship between the PGC-1α and CS activity as an indirect marker of mitochondrial biogenesis was observed. Because of this, we believe that a partly simplified explanation in our recent work is acceptable. Nevertheless, further investigations are necessary.

## Figures and Tables

**Table 1:** The corticosterone (CORT) concentration in the plasma.

	SHM (*n* = 6)	ST2 (*n* = 6)	ST4 (*n* = 5)
CORT (ng/mL)	88.6 ± 45.6	170.1 ± 104.1 ***	445.9 ± 31.6 ^###^

Results were expressed as mean ± SD, *** *p* < 0.001 ST2 vs. ST4, ^###^ *p* < 0.001 SHM vs. ST4.

## Abbreviations

BST	Bed nucleus of the stria terminalis
CAT	Catalase
CORT	Corticosterone
Cu/ZnSOD	Copper/zinc-dependent superoxide dismutase
CS	Citrate synthase
CSR	Chronic stress response
DEX	Dexamethasone
DH-CORT	11-dehydrocorticosterone
GCs	Glucocorticoids
GPx	Glutathione peroxidase
GR	Glucocorticoid receptor
GSSG	Glutathione disulfide
HSD11B1	11β-hydroxysteroid dehydrogenase type 1
HSD11B2	11β-hydroxysteroid dehydrogenase type 2
IGF-1	insulin-like growth factor 1
MDA	Malondialdehyde
MR	Mineralocorticoid receptor
MnSOD	Manganese-dependent superoxide dismutase
ROS	Reactive oxygen species
SH	Sulfhydryl groups
